# Évaluation Externe de la Qualité de la Goutte Épaisse/Frottis Sanguin pour le Diagnostic du Paludisme dans les Districts Sanitaires de Lomé et du Golfe au Togo

**DOI:** 10.48327/S1SQ-3476

**Published:** 2021-02-18

**Authors:** A.M. Dorkenoo, K.C. Kouassi, Y.-G. Afanyibo, K. Gbada, K. Yakpa, M. Têko, A.K. Koura, G. Katawa, M. Adams, M. Merkel

**Affiliations:** 1 Faculté des sciences de la santé, Université de Lomé-Lomé, Togo; 2 Ministère de la santé et de l'hygiène publique-Lomé, Togo; 3 Ecole supérieure des techniques biologiques et alimentaires, Université de Lomé-Lomé, Togo; 4 Institut national d'hygiène-Lomé, Togo; 5 Programme national de lutte contre le paludisme-Lomé, Togo; 6 Université de Kara, Togo; 7 Global Scientific Solution for Health, Maryland, USA

**Keywords:** EEQ, Goutte épaisse-frottis sanguin, Paludisme, Performances, contrôle de qualité, laboratoires, Lomé, Togo, Afrique intertropicale, EQA, Thick-thin blood smear, Malaria, Performances, Quality control, Laboratories, Lomé Togo, Sub-Saharan Africa

## Abstract

**Objectifs:**

Cette étude a pour but d'évaluer la performance de la goutte épaisse/frottis sanguin aux fins d'amélioration de son processus de réalisation.

**Matriel et Mthodes:**

Il s'est agi d'une étude transversale descriptive réalisée de mai à juin 2017 et portant sur des laboratoires relevant des secteurs public, libéral et confessionnel à Lomé, évalués sur le diagnostic parasitologique du paludisme. Une série de 13 lames de goutte épaisse/frottis sanguin de densités parasitaires variables avec des valeurs assignées (VA) de densité parasitaire et l'espèce plasmodiale affectées, a été utilisée. Le critère de conformité de la densité parasitaire était VA ± 25% et les taux de performance ont été comparés aux 80% recommandés par l'OMS-AFRO.

**Résultats:**

41,9% (13/31) des laboratoires participants avaient un taux de conformité supérieur à 80% dont 4 avec une performance de 100% pour l'aptitude à déterminer l'espèce plasmodiale. Pour les parasitémies < 100/μl, 51,6% des laboratoires participants avaient un taux de performance inférieur à 80% alors que 100% de ces laboratoires avaient un taux de performance supérieur à 80% pour les parasitémies > 2000/μl.

**Conclusion:**

Les laboratoires évalués avaient des capacités insuffisantes pour l'identification de *Plasmodium falciparum* et l'estimation correcte des faibles parasitémies. Une nécessité de renforcement d'aptitudes techniques, adaptées au contexte de faible parasitémie s'impose pour améliorer la confirmation biologique du paludisme au Togo.

## Abstract (en)

**Objective:**

In sub-Saharan Africa where 90% of malaria cases are concentrated, the control of this disease constitutes a major challenge whose diagnosis by thick and thin smear deserves to be exact and reproducible. The purpose of this study is to assess the performance of thick/thin blood smear in order to improve its implementation process.

**Material and methods:**

This was a descriptive and analytical study that took place from May to June 2017 and involved participating laboratories (PL) coming from public, liberal and confessional sectors in Lomé. A set of 13 blood smear slides of variable parasite densities (PD) with assigned values (AV) of parasite densities and the *Plasmodium* species assigned was used. The criterion for establishing the parasite densities compliance interval was assigned values ± 25% and the performance rates were compared to the 80% recommended by the WHO for Africa region.

**Results:**

41.9% (13/31) of the PLs had a compliance rate greater than 80% including four with a performance of 100% for the ability to identify the *Plasmodium* species. For the parasitaemia < 100/μl, 51.6% of participating laboratories had a performance rate less than 80% and for parasitaemia > 2000/μl, 100% of these laboratories had a performance rate greater than 80%.

**Conclusion:**

The evaluated laboratories had insufficient ability for the identification of *Plasmodium falciparum* and the correct estimation of low parasitaemia. A need to strength the technical skills, adapted to the context of low parasitaemia are essential to improve the biological diagnosis of malaria in Togo.

## Introduction

Au Togo, le paludisme qui sévit de façon endémique avec des recrudescences saisonnières, demeure un problème majeur de santé publique [4, 8, 12]: en effet la mortalité spécifique palustre est passée de 0,3 ‰ en 2011 à 0,12 ‰ en 2016 tandis que la létalité est passée de 6,5% à 3,92% chez les enfants de moins de 5 ans sur la même période [15].

La méthode de référence pour le diagnostic biologique de cette parasitose demeure la goutte épaisse/frottis sanguin (GE/FS), qui doit être exacte et reproductible [3, 11]. La norme ISO 15189 [6] exige une vérification périodique de cette exactitude par la participation à des programmes nationaux ou internationaux d'évaluation externe de la qualité (EEQ) selon la norme ISO 17043 [5]. C'est dans cette optique que la Division des laboratoires du Togo, a conduit en 2017, une campagne d'EEQ du diagnostic parasitologique du paludisme qui s'est fixée comme objectif d'évaluer la performance de l'examen de GE/FS aux fins d'amélioration de son processus de réalisation.

## Matériel et Méthodes

Il s'est agi d'une étude transversale descriptive et analytique qui s'est déroulée du 8 mai au 30 juin 2017 en quatre étapes: réception des échantillons et sélection des laboratoires participants (LP); validation des valeurs assignées et établissement des critères de performance; envoi des échantillons aux laboratoires participants, réalisation des essais et collecte des résultats et informations associées; exploitation des données et rendu des résultats avec proposition de quelques pistes de mesures correctives aux laboratoires participants.

### Réception des échantillons et sélection des laboratoires participants

Une série de 13 lames de goutte épaisse et frottis sanguin (ou frottis sanguin) (GE/FS) préparée par les CDC d'Atlanta, a été fournie à la Division des laboratoires pour cette évaluation. La GE et le frottis sanguin ont été confectionnés sur chaque lame qui a été colorée au Giemsa puis lutée. Des valeurs assignées de densités parasitaires (DP) variables ont été affectées à chaque lame positive, accompagnées de l'espèce plasmodiale à identifier.

**Fig. 1:**
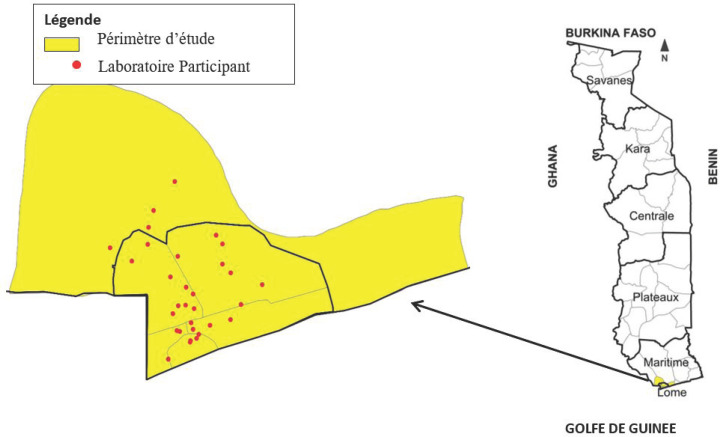
Répartition géographique des laboratoires participants Geographical distribution of participating laboratories

Trente-deux laboratoires de tous les secteurs des 5 districts de la région sanitaire de Lomé-commune et du district du Golfe couvrant l'aire géographique du Grand Lomé la capitale, relevant des trois niveaux du système sanitaire pyramidal et ayant un taux de fréquentation élevé, ont été sélectionnés par convenance pour cette évaluation (Fig. 1) [1].

Chaque laboratoire évalué a été anonymisé par attribution de lettres P, PV et C suivi du numéro d'ordre, ces laboratoires relevant respectivement des secteurs public, libéral et confessionnel.

### Méthode de calcul des densités parasitaires et gamétocytaires

Pour toute GE/FS positive, les DP et densités gamétocytaires (DG) étaient calculées en comptant parallèlement le nombre de leucocytes et de parasites conformément aux procédures standardisées décrites dans le manuel de réalisation de la GE/FS élaboré par le PNLP du Togo [14]. Le nombre de parasites était compté pour 200 globules blancs (GB) ou 500 parasites contre le nombre de GB comptés. Ces densités étaient exprimées respectivement en parasites asexués/μl de sang et gamétocytes/μl de sang selon la formule [10]:

$$\text{DP}=\frac{\text{Nombre}\left(\text{x}\right)\text{de parasites}*\text{8000}}{\text{Nombre}\left(\text{y}\right)\text{de globule blancs}}\left(\text{parasites}/\mu l\right)$$

### Valeurs assignées et critères d'acceptabilité

Quatre performances ont été définies pour être évaluées pour chaque laboratoire participant: deux performances qualitatives (détermination de la négativité ou de la positivité des lames et reconnaissance de l'espèce plasmodiale) et deux quantitatives (DP et DG).

### Valeurs assignées

Les performances des laboratoires étaient évaluées par rapport aux valeurs de référence assignées par les CDC qui ont été ensuite confirmées par le laboratoire de référence (LR) du PNLP du Togo. En effet, les valeurs assignées (VA) des différentes lames envoyées par CDC (VACDC) ont été confirmées par un microscopiste accrédité N°2 de l'OMS, par double lecture de chaque lame. La moyenne des parasitémies pour chaque lame positive correspondante à une médiane et l'intervalle d'acceptabilité à cette médiane à ± 2DMA (Déviation Médiane Absolue) ont été calculés. L'intervalle d'acceptabilité associé aux valeurs assignées a été déterminé en appliquant ± 25% (limite de variabilité recommandée par l'OMS) aux densités parasitaires [11].

Pour qu'une différence soit non significative entre les valeurs assignées par les CDC et celles du laboratoire de référence (VA_LR_), une erreur normalisée (EN) déterminée entre les deux valeurs devrait être conforme, correspondant à une valeur comprise entre -1 et 1. Selon la norme ISO 13528, cette erreur est déterminée par la formule [7]:

$${\left(E_{n}\right)}_{i}=\frac{x_{i}-x_{pt}}{\sqrt{U^{2}\left(x_{i}\right)+U^{2}\left(x_{pt}\right)}}$$

X_pt_ était la valeur assignée déterminée dans un laboratoire de référence,

X_i_ était la valeur fournie par le CDC

U (x_i_), l'incertitude élargie correcte devant affecter la valeur X_i_, qui était de ± 25% de la valeur assignée de CDC.

U (x_pt_) correspondait à l'incertitude élargie devant affecter la VA_LR_ soit VA_LR_ ± 25%.

Le laboratoire de référence a également déterminé la gamétocytémie pour les lames sur lesquelles les gamétocytes ont été identifiés de même que leurs intervalles d'acceptabilité calculée, en appliquant les ± 25% aux valeurs assignées obtenues. Le CDC n'avait pas fourni de valeurs assignées pour la densité gamétocytaire.

### Critères d'acceptabilité

Des critères d'acceptabilité pour les quatre niveaux de performances ont été établis: l'aptitude de reconnaissance de la positivité/négativité d'une lame; l'aptitude de reconnaissance des différentes espèces plasmodiales; l'aptitude pour l'estimation des densités parasitaires et gamétocytaires. À été considéré comme acceptable pour un laboratoire participant, un résultat de ces densités dans l'intervalle de conformité déterminé par VA_LR_ ± 25%:

- aptitude de reconnaissance de la positivité/négativité d'une lame, déterminée par le taux de conformité (TC) de reconnaissance de lames POS/NEG

$$=\frac{\text{Normbre lames rsutat correct}}{\text{Nombre total de lames reues}}x100$$

- aptitude d'identification des espèces, déterminée par le taux de conformité d'identification d'espèce plasmodiale

$$=\frac{\text{Normbre lames rsutat correct d'espce}}{\text{Nombre total de lames reues}}x100$$

- conformité des densités parasitaires, déterminée par le taux de conformité d'estimation de la densité parasitaire (DP)

$$=\frac{\text{Normbre de lames avec densit parasitaire correcte}}{\text{Nombre total de lames reues}}x100$$

- conformité des densités gamétocytaires déterminée par le taux de conformité d'estimation de la densité gamétocytaire (DG)

$$=\frac{\text{Normbre de lames avec densit gamtocytaire correcte}}{\text{Nombre total de lames reues}}x100$$

### Dispatching des échantillons et essais

Chaque laboratoire participant a reçu un lot de 6 lames, constitué de 2 négatives et de 4 positives à densité parasitaire et gamétocytaire variables, y compris une lame de parasitémie < 100 parasites/μl. Ils ont disposé de 3 jours pour réaliser l'essai et rendre les résultats.

### Collecte des données, analyses statistiques et compte rendu d'EEQ

Les données recueillies sur un formulaire élaboré à cet effet ont été analysées par le logiciel Access 2016 (Microsoft, USA). Les taux de conformité, moyenne et écart-type ont été déterminés. Le taux cible de résultats conformes attendu était de 80% pour chaque performance conformément à la recommandation des systèmes d'évaluation SLIPTA (Stepwise Laboratory Process Toward Accreditation) de l'OMS-AFRO [13].

## Résultats

### Valeurs assignées, intervalles d'acceptabilité

Les valeurs assignées par les CDC étaient confirmées par le laboratoire de référence, y compris les lames (1046, 1047, 1048 et 1049) dont la DP assignée était nulle. Les gamétocytes étaient identifiés par le laboratoire de référence sur les lames 1033 (60 ± 15 gamétocytes/μl) et 1041 (28 ± 7 gamétocytes/μl). Le tableau 1 résume les niveaux cibles de performances attendues.

**Tableau I:** Valeurs assignées avec intervalles d'acceptabilité utilisés Assigned values with acceptability intervals used

N° Lames GE	Parasitémie (p/µl)		EN VA_LR_ vs VA_GHSA_	Espèce plasmodiale
	VA_LR_ ± 25% VA	VA_GHSA_ ± 25% VA/2MAD		
1031	213 ± 53	208 ± 52/96	0,1	*P. falciparum*
1032	1300 ± 325	1232 ± 308/416	0,2	*P. falciparum*
1033	2356 ± 589	2496 ± 624/400*	0,2	*P. falciparum*
1034	134 ± 34	96 ± 24/64	0,9	*P. falciparum*
1036	214 ± 53	208 ±52/96	0,1	*P. falciparum*
1038	619 ± 155	616 ± 154/256	0,0	*P. falciparum*
1041	572 ± 143	616 ± 154/256	0,2	*P. falciparum*
1043	98 ± 24	96 ± 24/64	0,0	*P. falciparum*

### Niveau de performance des laboratoires à détecter la présence de *Plasmodium* sp

**Fig. 2:**
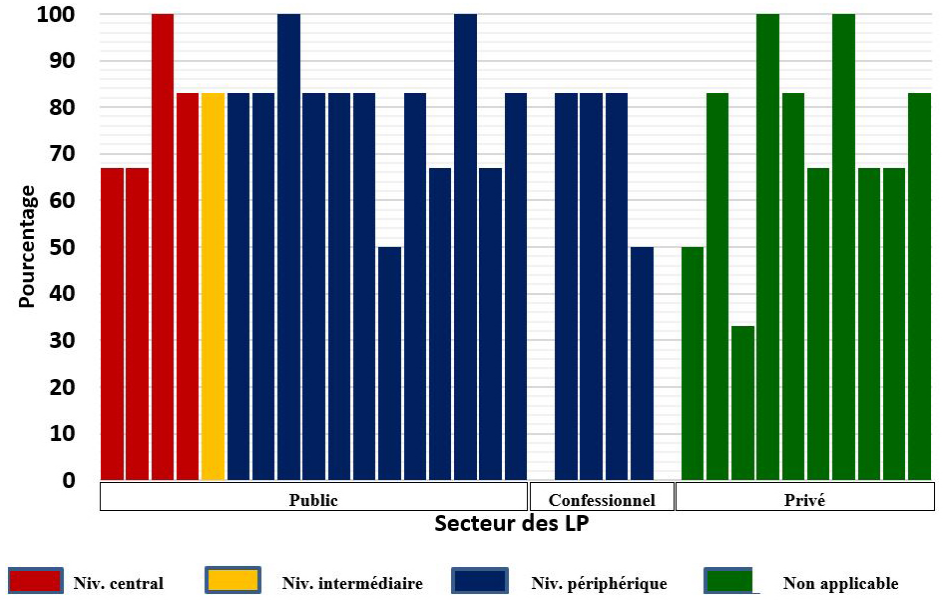
Taux de conformité par rapport à la positivité Compliance rate by slides positivity

Le taux de participation des laboratoires évalués était de 97%, un des 32 laboratoires sélectionnés n'ayant pas rendu ses résultats. De ces 31 laboratoires, 64,5% (20/31) avaient un taux de conformité supérieur à 80% dont 5 avec une performance de 100%. Ceux du niveau central du secteur public ont obtenu un taux de conformité de 33,3% (1/3) alors que ceux de niveau périphérique du secteur public, une performance globale de 75,0% (9/12) (Fig. 2).

### Performance des laboratoires participants par rapport à l'aptitude à estimer correctement la parasitémie et la gamétocytémie

**Fig. 3:**
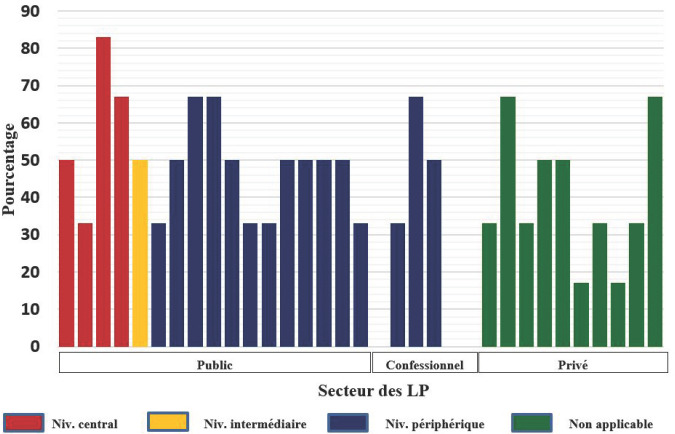
Taux de conformité par rapport à la densité parasitaire Compliance rate by parasite density

Un taux de conformité supérieur ou égal à 50% pour l'estimation de la parasitémie était noté pour 74% (23/31) des laboratoires participants dont un seul (3,2%) avec un taux de 100% (Fig. 3). Ce taux qui de façon générale était de 100% pour les parasitémies fortes, a chuté à 51,6% pour les parasitémies faibles (Tableau 2). Pour l'estimation de la densité gamétocytaire, aucun cas de conformité n'était noté.

**Tableau II:** Taux de conformité par niveau de parasitémie des lames Compliance rate by slides parasitaemia level

Parasitémie ou densité paasitaire (DP) (/µl)	Nombre de lectures	Nombre de conformités	%
Faible (DP < 100)	31	16	51,6%
Moyenne (100 ≤ DP ≤ 2000)	78	62	79,5%
Élevée (DP > 2000)	15	15	100%

### Performance des laboratoires pour l'identification de l'espèce plasmodiale

**Fig. 4:**
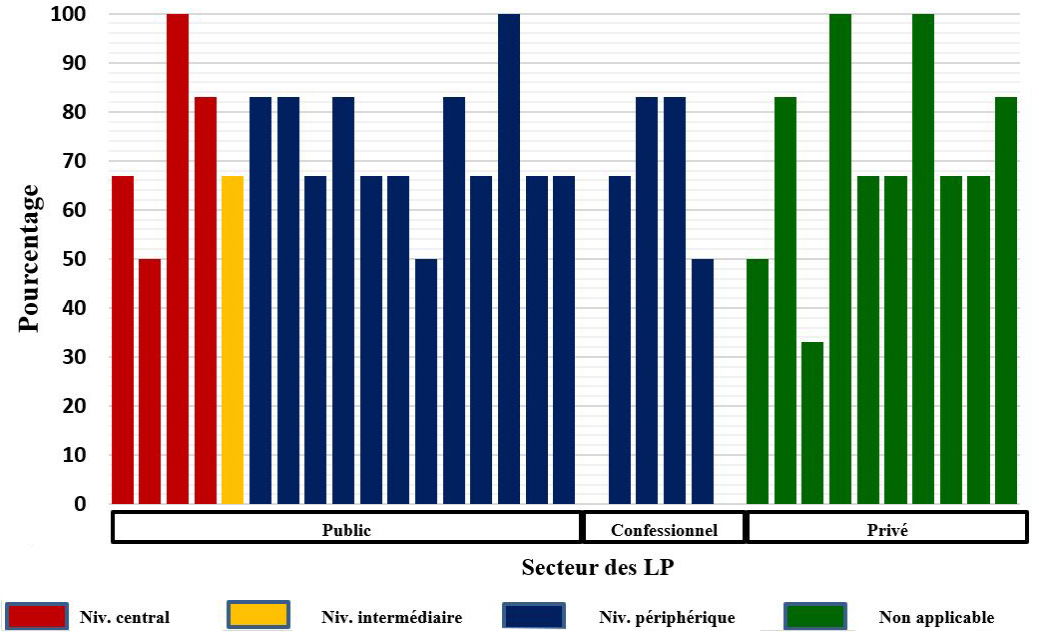
Taux de conformité par rapport à l'identification de l'espèce plasmodiale Compliance rate by Plasmodium species identification

Près de 42% (13/31) des laboratoires évalués avaient un TC global supérieur à 80% dont 4 avec un taux de conformité de 100% pour l'identification de l'espèce plasmodiale. Les extrêmes étaient de 33,3% et 100,0% (Fig. 4).

## Discussion

L'OMS recommande la GE/FS et les tests de diagnostic rapide (TDR) pour la confirmation biologique de cas suspects de paludisme. La présente étude qui n'a couvert qu'une des 6 régions sanitaires que compte le pays, a permis de jeter les bases d'un Programme national d'évaluation externe de la qualité du diagnostic parasitologique par la GE/FS au Togo [2].

Le critère de valeurs assignées et intervalles d'acceptabilité établis par le laboratoire de référence national a été préféré au Z-score comme critère de jugement dans notre étude, car seules 8 des 13 lames utilisées disposaient de valeurs assignées quantitatives.

Cette option a été également faite au Pakistan en 2007 par Muhammad et al [2]. En effet la formule de validation des valeurs assignées et l'erreur normalisée utilisée était plus adaptée que la simple comparaison de valeurs ou de moyennes au seuil de p < 0,05, car elle prend en compte les variabilités acceptables (±25% VA) recommandées par l'OMS. La variabilité ± 2 MAD fournie par le CDC Atlanta était dans 87% des cas supérieure à celle obtenue avec ± 25% VA [16].

Pour chaque LP, le taux de conformité pour tous les 4 niveaux de performance évalués, devait être supérieur à 80% comme le recommande l'OMS-AFRO [13]. Mais la performance globale de 64,5% pour l'aptitude à identifier l'espèce plasmodiale de notre étude dénote une capacité insuffisante de l'ensemble des LP à correctement reconnaitre *P. falciparum* sur les frottis sanguins, malgré les nombreuses sessions de mise à niveau et de supervisons formatives régulièrement organisées par le PNLP à l'intention des techniciens de laboratoire avec l'appui du Fonds mondial pour le sida, la tuberculose et le paludisme. Toutefois, la fiabilité d'un résultat de GE/FS n'étant pas uniquement dépendant de l'expertise du microscopiste, l'état des équipements, en l'occurrence les microscopes binoculaires et la qualité des réactifs de coloration et de fixation utilisés dans ces LP, pourraient influer les résultats obtenus. L'utilisation des outils de contrôle interne de la qualité, du manuel de l'OMS sur l'assurance qualité en microscopie du paludisme pourrait aider ces laboratoires à améliorer le niveau de leur performance [11]. En effet, un programme d'EEQ portant sur 8118 lames au Pakistan en 2007 a utilisé des modules de formation élaborés à partir des objectifs du document OMS pour améliorer la performance des laboratoires évalués et détecter la valeur ajoutée du programme EEQ.

Les LP des secteurs confessionnels, du service de santé des armées et du niveau périphérique du secteur public dans notre étude, semblent obtenir de meilleures performances; constatation qui pourrait s'expliquer par la compétence acquise par le personnel de ces laboratoires suite à une pratique plus soutenue pendant de nombreuses années. Quant à la performance exceptionnelle de 100% de 5 des laboratoires participants de notre étude, un de niveau central, 2 de niveau périphérique et 2 du secteur libéral, elle s'expliquerait par une application beaucoup plus rigoureuse des procédures d'une microscopie de qualité lors de la réalisation de cet examen. Notre taux de performance globale était toutefois supérieur aux 46,7% retrouvés par Getachew et al lors d'une évaluation de 30 laboratoires publics de 2014 à 2015 en Éthiopie [16].

Dans notre étude, les LP ont eu plus de facilité à déterminer les fortes parasitémies (TC de 80% pour tous les LP) que les faibles (TC de 80% pour 3,2% des LP). Cette performance faible pour l'estimation de la parasitémie démontrerait le non-respect de toutes les étapes de la procédure de réalisation par les LP. En effet, pour les faibles parasitémies, l'OMS recommande de compter plus de 500 leucocytes avant d'estimer la parasitémie [10], mais peu de techniciens de laboratoire le font, arguant une charge de travail journalière élevée. Or cette insuffisance à correctement estimer ces parasitémies faibles, pourrait impacter négativement la prise en charge thérapeutique correcte de ces cas, sachant que notre aire d'étude située en zone urbaine, est caractérisée par un faible niveau de circulation plasmodiale. Une évaluation similaire conduite dans 445 laboratoires publics de 2013 à 2014 en République démocratique de Congo, avait retrouvé un résultat acceptable dans 32,5% des laboratoires [9], loin des 51,6% de notre étude.

Face à ce constat des limites de la GE/FS pour la confirmation optimale du paludisme dans les pays endémiques à faibles ressources, il serait sans doute judicieux de positionner les TDR pour le diagnostic biologique du paludisme, même dans les zones avec microscopie. Mais en attendant que l'OMS ne propose de nouvelles recommandations allant dans ce sens, les partenaires des PNLP devraient appuyer les pays à conduire régulièrement les activités d'EEQ pour les aider à relever le niveau de la confirmation biologique de cette parasitose [11].

## Conclusion

La mise en oeuvre d'un programme national d'EEQ dans les pays à ressources limitées, bien que difficile, demeure indispensable pour l'application des exigences de l'OMS en microscopie de qualité pour le diagnostic biologique du paludisme. Cette étude qui a mis en exergue la faible capacité des laboratoires évalués à correctement identifier les espèces plasmodiales et à estimer la parasitémie, implique la nécessité d'aller au-delà des formations continues habituelles pour identifier des approches novatrices pour suppléer cette méthode diagnostique largement utilisée mais qui présente de nombreuses insuffisances.

Cette évaluation de la GE/FS limitée à une seule région, devra être étendue à l'échelle nationale, mais également aux TDR pour un diagnostic biologique davantage fiable du paludisme dans les pays endémiques.

## Remerciements

Nos remerciements au Ministère de la santé et de l'hygiène publique, aux CDC d'Atlanta à travers le projet GHSA pour l'appui financier, à l'ONG GSSH pour sa collaboration, au Programme national de lutte contre le paludisme (PNLP), à tous les membres de l'équipe du Programme national d'évaluation externe de la qualité de la DL et aux laboratoires participants.

## Conflits D'intérêts

Les auteurs ne déclarent aucun conflit d'intérêts.
